# Does Experience of Failure Decrease Executive, Regulatory Abilities and Increase Aggression? Affective-Cognitive Regulation of Aggressive Behaviors

**DOI:** 10.5539/gjhs.v4n6p60

**Published:** 2012-08-30

**Authors:** Farzaneh Pahlavan, Christophe Mouchiroud, Emna Nemlaghi-Manis

**Affiliations:** 1 Laboratoire de Psychologie Sociale, Université Paris Descartes, Paris, France; 2 Laboratoire d’Adaptation de Travail Individuel, Université Paris Descartes, Paris, France

**Keywords:** aggression, self-regulation, ECF, DES, health care workers, success/failure paradigm

## Abstract

Recent advances in the study of affective-cognitive regulation of aggressive behavior suggest positive correlations between poor executive capacities (ECF) and dispositional negative reactivity (Posner & Rothbart, 2000). If the global assumption is correct what are the likely implications of predicted relation? The central issue in present research was to verify this assumption and examine how situational characteristics could alter executive performance in persons with Dysexecutive Syndrome (DES, Baddeley, 1998) and healthy adults (students, health workers) to explore some of the consequences of those modifications for aggressive tendencies. Precisely, we expected the positive correlations between poor executive performances and high aggressive tendencies at dispositional as well situational levels, except for health workers, given their professional duties.

In order to assess cognitive capacities and dispositional as well as situational aggressive tendencies, during two studies (First study: N=60 students; Second study: N= 60 students, N= 24 patient with Dysexecutive Syndrome, N= 45 health care workers) right-handed French-speakers participants completed twice, during an initial phase of the study and one week after, a series of standard executive functions neuropsychological tests and aggression questionnaires. During second phase, participants executed a task introducing the experimental feedbacks (success, neutral, failure) before completion of neuropsychological tests and questionnaires. The results provided evidence of a dispositional relationship between poor executive functioning and aggressive tendencies, and extended it to situational level. For all participants, it showed that increases in impulsiveness (negative emotionality and aggressive choices) due to a negative feedback were concomitant with an inability to focus individuals’ attention on ongoing tasks.

## Part I. Self-regulatory Processes and Executive Cognitive Functions: Literature Revision

Self-regulatory mechanisms refer to many processes by which the human psyche exercises control over its functions, states, and inner processes (Vohs & Baumeister, 2004). Defined as capacities of individuals to symbolically plan, represent, and control their thoughts and behaviors, self-regulation is emphasized to be fundamental to intrapersonal and interpersonal behavioral adjustment (Forgas, Baumeister, & Tice, 2009). Developed during interaction between the child and his/her social environment (e.g. parents, peer), as a part of socialization process, all normal people are supposed to have regulatory capacities, including a strong subjective feeling of intention or voluntary control of their behavior (Posner & Rothbart, 2004). Therefore, much of so called self-regulation in everyday terms involves conscious, effortful, and motivated activities by which individuals try to bring their thoughts and behaviors into accord with consciously desired standards (e.g. Oettingen & Gollwitzer, 2009). It is also clear that many of the dynamic processes involved (e.g. spontaneous switching between multiple goals) are automatic and unconscious (e.g. von Hippel & Ronay, 2009). Thus, for theoretical and practical purposes, as Vohs and Baumeister (2004) we propose to define self-regulation as any conscious as well as unconscious processes involved in regulation and control of any human inner states or responses, including thoughts, emotions, impulses, performance, as well as attention.

Working on attentional regulatory processes, Posner and colleagues try to bring a flexible and dynamic view of the self-regulation mechanisms and propose to distinguish three attention networks in the brain serving different functions and having different neural anatomies (e.g. Posner, 1980; Fan & Posner, 2004; Rueda, Posner, & Rothbart, 2004). These networks are emphasized to be the sources of attention and to operate in conjunction with other neuronal structures to regulate basic cognitive and emotional processes, with which people cope in their everyday existence (Eisenberg & Morris, 2002; Vohs et al., 2004). The first network controls *Altering* and provides achievement and maintenance of a state of high sensitivity to incoming stimuli involving frontal and parietal region of right hemisphere. The second network *Orienting* allows selection of sensory information and involves superior parietal cortex, temporal parietal junction, frontal eye fields, and superior colliculus. The third, the *Executive Attention* network functions to monitor and resolve conflict involving the anterior cingulated, lateral prefrontal cortex, and basal ganglia (Rueda et al., 2004). There is evidence that the prefrontal cortex, particularly on the right side, is involved in control through inhibition of the competing systems (e.g. Botvinick, Braver, Barch, Carter, & Cohen, 2001). The executive attention network provides, therefore, executive functions (Posner et al., 2004) in order to facilitate or inhibit the functions of other networks and to underlie self-regulation mechanisms (e.g. inhibition of an impulsive reaction to irrelevant stimuli while pursuing a cognitively represented goal; Posner & Rothbart, 1992, 1998, 2000). For example, one of the main neural structures of the executive attention network (the anterior cingulated gyrus), is linked to specific functions related to self-regulation (Davidson, Fox, & Kalin, 2007; Rothbart & Rueda, 2005) including the control of working memory, response to error, monitoring of conflict, and also regulation of emotion. In emotion studies, this neural structure is often seen as a part of a neural network (the orbital frontal cortex and amygdale) that regulates emotional responses to sensory information. Activation of this neural structure is observed whenever people are asked to control their automatic reactions to strong positive (Beauregard, Levesque, & Bourgouin, 2001) or negative (Ochsner, Bunge, Gross, & Gabrieli, 2002) emotions.

In her works focused on *Temperament* viewed as “constitutionally based individual differences in reactivity and self-regulation, in the domains of affect, activity, and attention” (Rothbart & Bates, 2006, p. 100), Rothbart (1989) distinguishes two major components of temperament, one associated with reactivity and the other related to voluntary self-regulation. In this view, reactivity refers to “responsiveness of emotional, activation, and arousal systems” whereas self-regulation is viewed as “approach, avoidance, and attention that modulate reactivity” in function of social demands of situation (Rothbart, Ahadi, Hersey, & Fisher, 2001). Eisenberg’s model of emotion-regulation (Eisenberg et al., 2009; Eisenberg et al., 2004; Eisenberg et al., 2002) differentiates also between reactive control and executive control. However, in her perspective, only the executive control is truly part of emotion regulation. Accordingly, in contrast to executive controls, reactive controls are so automatic that they often are not under voluntary control (Eisenberg et al., 2009).

Thus, conceptually, it is conceivable to expect both cognitive and reactive controls to be involved in individuals’ behavioral regulation because behavioral problems (e.g., externalizing problems such as aggression) are often defined partly in terms of problems in controlling emotion (e.g., displaying high level of anger or anxiety). Nevertheless, some forms of behavioral problems, involving disorders of affect, may be best conceptualized as disorders of the context regulation of affect (Davidson et al., 2007). That is, the emotion characterizing these disorders would be normative and appropriate in certain contexts. For example, anxiety disorders typically involve the expression of normal emotion in inappropriate or non-normative contexts. Therefore, appropriately contextual regulation of emotional behavior might involve both reactive and cognitive controls.

Indeed, the role of context in terms of executive functions and affective regulation is relatively understudied. For Davidson, Fox, and Kalin (2007) the contextual regulation of emotion is assumed to proceed relatively automatically. Thus, regulation of emotion may sometimes demand precedence over other aspects of self-regulation. However, emotions do not uniformly affect individuals’ self-regulatory capacities. While some emotions (both positive and negative) can cause individuals to ignore relevant information, other emotions may mobilize their attentional capacities. Failure to allocate attention and to regulate cognitive processes in order to incorporate all the relevant information, may contribute to self-defeating behavior as well as to unrealistic vision of the social world (Dodge & Pettit, 1990). In the same line, the acute bad feelings may stimulate the desire to make them stop to a degree that can cause self-regulation failure in the long run (Baumeister, Zell, & Tice, 2007). In all cases the individuals apprehend context based on their analysis of current context, and they try to adjust their behavior in an apparently appropriate way regarding current context. Thus, study the role of context in affective and cognitive processes could lead to discover different causal pathways leading to the self-regulation success or failure.

## 1. Age-related Differences in Executive and Reactive Controls of Behavioral Adjustment

In Rothbart’s approach, self-regulation is considered as a major part of developmental organization of temperament and personality, which follows a course of development and coincides with increased ability to regulate internal states and reactions to external events. Some of these self-regulatory mechanisms accompany the maturation of attentional mechanisms (Posner & Rothbart, 2000).

Rapid development of executive attention, during the first and second years of life, allows development of volitional skills, which by practice become less controlled and more automatic (Rothbart et al., 2004). Thus, executive changes over time should be due to environmental influences and socialization, suggesting a fundamental continuity in development, with social and emotional development at childhood laying the groundwork for adult functioning (Eisenberg et al., 2005; Nigg, 2006; Rothbart & Bates, 2006).

In general, behavior problems are characterized by negative reactivity, specifically by an anger bias. This may take the form of a high sensitivity to anger-related appraisals and mal-adaptive responses (aggression) to anger-eliciting situations (Hubbard et al., 2002). High negative reactivity could diminish capacity to attend social cues, leading to misinterpretation and incorrect processing of social information (e.g. Hostile attribution bias; Crick & Dodge, 1996), with the risk for psychopathology such as externalizing disorders (Kazdin, 1995). Indeed, externalizing/internalizing behavioral problems are based on individuals’ reactions to stressors. Externalizing behaviors are primarily characterized by actions such as antisocial behavior, hostility, and aggression which may, in some cases, coincide with the inhibitory problems of attention-deficit/hyperactive disorder (ADHD; Mullin et al., 2007). Internalizing behaviors are primarily characterized by processes within self, such as anxiety, somatization, depression, and withdrawal (American Psychiatric Association, 2000). However, while association between executive controls and internalizing problems is relatively unclear (Eisenberg, Hofer, & Vaughan, 2007, p. 297), there is mounting evidence relating deficits in executive control to children’s externalizing problems (e.g., Eisenberg et al. 2009; Kochanska & Knaack, 2003; Lengua, 2006; Oldehinkel, Hartman, Ferdinand, Verhulst, & Ormel, 2007; Spinrad, Eisenberg, Gaertner et al., 2007). Nevertheless, it seems that only deficits of some aspects of executive control, such as inhibitory control, were related to externalizing problems, whereas planning was not (Martel, Nikolas, & Nigg, 2007).

In sum, there is noticeable evidence of typically association between externalizing problems and negative emotionality such as anger, frustration, and irritability (Eisenberg et al., 2009; Gilliom, Shaw, Beck, Schonberg, & Lukon, 2002; Lengua, 2006; Oldehinkel et al., 2007; Zeman, Shipman, & Suveg, 2002; Rothbart et al., 2006). Such feelings may motivate externalizing behaviors, and externalizing children, especially if aggressive, may become more angry and hostile over time because they tend to be rejected and victimized by peers (Rubin, Bukowski, & Parker, 2006). However, the question regarding stability or variability of these relations at older ages is not established yet.

## 2. Gender-related Differences in Executive and Reactive Controls of Behavioral Adjustment

There is therefore evidence suggesting age as moderator of relation between behavioural problems and the development of executive control. However, there is debate about the moderator role of gender. In terms of general cognitive abilities, despite differences noted on specific ability tests, males and females seem do about the same. In addition, analyses of trends over time suggest that gender differences in scores on cognitive ability tests decrease over the years (Feingold, 1988). There is only some agreement in regard to the eventual effects of the differences in information processing (such as attention and perception). In fact, researchers have consistently found that males show an advantage in visual-spatial abilities (Halpern, 1997; 2000; Kimura, 1992; Weiss, Kemmler, Deisenhammer, Fleischhacker, & Delazer, 2003; Overman, Bachevalier, Schuhmann, & Ryan, 1996; Shaywitz et al., 1995), and females show an advantage for verbal abilities (Halpern, 2000; Kimura, 1992; Krüger, Krüger, Hugo, & Campbell, 2001; Carlson & Moses, 2001). Thus, many researchers emphasize that there are more similarities than differences between the cognitive and affective abilities of males and females (see Sternberg, 2004). There is also much variability within gender groups, particularly for males (Halpern, 2000), males are over-represented, with higher percentages identified as having disabilities, including dyslexia, delayed speech, and attention deficit/hyperactivity disorder, as well as giftedness. However, there is also evidence that methodological bias plays a role in undercounting females with dyslexia (Smith, Kimberling, & Pennington, 1991), as well as females who are gifted. For Stenberg (2004), gender differences refer only to average and they may originate from a number of factors, many of which change over time.

Behaviourally, because of the higher prevalence of serious externalizing problems among males than females, most of the extant literature in this area has been conducted with male sample. Thus, it is not clear whether links between poor emotion regulation (anger burst) and externalizing behaviour (particularly reactive aggression) apply similarly to females. In females, research has documented significantly lower rates of overt aggression, but higher rates of relational aggression (for example, indirectly retaliating against a peer by gossip), suggesting, among others, a methodological bias of traditional studies of aggression (Bettencourt & Miller, 1996).

## Part II. Present Research

As an obvious feature of normal socialization, and in sense of resisting temptation (voluntarily control of current impulses for the sake of expected future benefits), self-regulation capacities could be therefore of specific interest to understand social behaviors such as aggression. Indeed, there is growing evidence that individual differences in cognitive functioning is a risk factor for aggressive behavior at dispositional level, that is some people’s behavior may be chronically influenced by impulsive processes across time and situations (e.g. Eisenberg et al., 2009; Fishbein, 2000; Hawkins & Trobst, 2000; Morgan & Lilienfeld, 2000; Paschall & Fishbein, 2002; Giancola, 2004; Giancola & Mezzich, 2000). However, one of the important challenges to better understanding self-regulation of aggressive behavior is to determine implication and role of different contexts in activating affective and cognitive processes leading to such behavioral choices. Only few studies have directly focused on situational differences in aggression related to higher order regulatory processes in terms of executive functions (e.g. DeWall, Baumeister, Stillman, & Gailliot, 2007; Tangney, Baumeister, & Boone, 2004). If aggressive tendencies depend on chronic or contextual alteration of executive functions, the improvement of executive controls by training programs should be of utmost importance for patients with psychopathology such externalizing disorders or with brain injury associated with Dysexecutive Syndrome (DES) as well as for professional such as health care workers with the risk of burnout.

As a matter of fact, the existing literature in these area shows that in spite of the early-development of the executive attention network (between ages 2 and 7), with practice, the ability to self-regulate can be trained and improved. Actually, recent training programs have resulted in improved executive control and behavioral adjustment within special population (patient with specific brain injury; Sohlberg, McLaughlin, Pavese, Heidrich, & Posner, 2000; ADHD, Kerns, Eso, & Thomson, 1999; Semrud-Clikeman, Nielsoen, & Clinton, 1999; Klingberg, Forssberg, & Westerberg, 2002) and domains (attention process, working memory), even in animal (Rumbaugh & Washburn, 2003).

The present research was designed to test the link between executive functions, and chronic/contextual aggressive tendencies using a feedback paradigm, in general and clinical populations. The central issue was to examine whether contextual characteristics could alter executive controls, and to explore the behavioral consequences of these executive alterations. We focused on a variety of executive functions thought to be relevant to control of attention, resistance to interference, error detection/correction, and response inhibition. Given the lack of critical pieces of information regarding the situational differences in aggression related to higher order regulatory processes, two two-part studies were conducted. The first part basically dealt with relatively stable individual differences linked to executive capacities and aggressive tendencies. The second part of the study would look at externally activated processes, primed by three specific task feedbacks (success, failure, neutral) that might contribute to depletion of executive capacities as well as augmentation of aggressive tendencies.

The first study was designed to assess in a student sample, (a) whether there was a positive relation of deficits in executive controls and aggressive tendencies, (b) if this relation was found before and after a negative feedback, (c) in men as well as women. The second study was a modified replication of the first one and examined the same issues in three different samples: a sample of students, a sample of hospital health care workers, and finally a sample of persons with Dysexecutive Syndrome (DES). The specific objective of the second study was focused on comparison of the data from non-clinical individuals (student and health care workers) to those of a clinical sample (persons with Dysexecutive syndrome; DES) in function of feedback conditions.

The case of DES is of great (instead particular) interest for our research (instead researcher) because of their particular emotional (difficulty for inhibiting many types of emotions such as anger, excitement, sadness, or frustration), cognitive (poor working memory and short term memory, difficulties to learn new information, impaired speech, reading comprehension, planning and reasoning), and behavioral (losing social skills because impaired judgments and insights into what others may be thinking) characteristics (Halligan, Kischka, & Marshall, 2004; Thornton, 2008). Dysexecutive syndrome (DES) results usually from brain damage to the frontal lobe. There is not one specific pattern of damage that leads to DES. People with DES can usually do well on the tests used to evaluate executive controls. However, their problems are related to integrating their capacities into everyday tasks (Wilson, Evans, Emslie, Alderman, & Burgess, 1998). According to studies published to date, many of the symptoms can be seen as a direct result of impairment of the central executive component of working memory, which is responsible for inhibitory control (Wilson et al. 1998). We believe that comparative studies of such cases might provide new perspectives on the relation between cognitive neuropsychology and social psychology in health sectors.

## A. First Study

Based on prior findings and the notion that negative emotionality involves executive control (e.g. Eisenberg et al., 2009; Mullin et al., 2007), we hypothesized that (Correlative Hypothesis) executive deficits would be positively correlated with aggressive tendencies, and these relations would be observed at a first assessment, and would hold at a second assessment, after participants received a feedback. That is, some individuals’ behavior may be chronically influenced more by impulsive processes independent of any experimental manipulations. However, (Causal Hypothesis) because executive control may be dynamically modulated by characteristics of current context, the effect of its deficits on aggressive tendencies was predicted to become stronger when participants received a negative feedback. Finally, our last hypothesis was gender related individuals’ differences. Given controversial issue regarding gender as moderator of executive functions, our gender-related hypotheses was based only on gender-differences regarding relations of experimental feedback and aggressive tendencies. More precisely, (Gender-related Hypothesis) we expected more aggressive behavioral intention in men and higher emotional negativity in women, particularly after receiving a negative feedback.

## 1. Method

### 1.1 Ethical Issues

We give careful consideration to the ethical issues surrounding the use of our experimental procedure in terms of feedback induction (failure/success). Given lack of a formal research Ethics Committee for non-clinical research projects involving human participants, we recruited only participants who agreed or were able to agree a consent form in accordance with the ethical principles of the American Psychological Association. In addition, we included participants who reported no recent traumatic events, informing them by consent form that they were free to withdraw at any time.

### 1.2 Participants

Sixty students of psychology (female=30, male=30) aged between 19 and 21 years (*M* = 19.88 years, *SD* = 0.72) from University Paris-Descartes participated in a two-session study. They were recruited via announcement put up around Faculty. Participants were all French speakers, and none reported any neuropsychological or developmental disorder. They participated on a voluntary basis, and after having read and signed a double informed consent form before starting and after finishing the experimental sessions. Participants were randomly assigned to one level of the experimental condition (feedback).

### 1.3 Experimental Conditions

There were three experimental conditions (Success, Failure, and Neutral) with a specific task feedback (negative, positive, neutral). To introduce the experimental feedback we used a modified version of a perceptual task devised by de Montmollin (1966). Participants were instructed that they should carefully look at a white page (A4: 210 X 297mm) covered by black spots (80 spots spread randomly) during 5 seconds and then estimate the number of the spots to a margin of 5% error. In addition, they were given statistical information about the average rate of success (12.5% for success condition) or failure (12.5% for failure condition) on this perceptual task. In the neutral condition statistical information indicated that generally people found the accurate response. The neutral feedbacks was designed to establish specific effect of affective feedbacks, and also to eliminate any putative hypothesis regarding involvement of eventual learning processes due to completion of the executive tests a second time.

The experimenter presented the white-spotted page for 5 seconds and then introduced the experimental feedback by means of three different messages, apparently based on the participant’s estimation. In the success condition, participants received a positive message: “Congratulations, you are among the 12.5% of subjects who completed the task with success. We will now continue.” A negative message was given in the failure condition: “Unfortunately, you are among the 12.5% of subjects who completed the task with failure. We will continue.” The control condition featured a neutral message: “Your answer is correct. This part of experiment is over. We will continue.”

## 2. Material and Measurement

### 2.1 Assessment of Executive Function

In order to evaluate executive capacities we applied part of paper/pencil French-version of a battery of worldwide standardized neuropsychological tests currently used by specialists in the field. The choice was based on the nature of the investigated executive functions and the time required for its completion. We chose the executive capacities of inhibition, initiation, planning, and selective attention or mental flexibility, which are currently used to assess inhibitory control (Moses & Carlson, 2004); and because our primary objective was to study effects of a specific emotion on executive performance, we selected tasks which required little time. For example, to evaluate capacity of planning, we used a Tower of Hanoi task with 4 disks. Performance indices were completion times, measured with a stopwatch. We chose executive capacities of inhibition (Stroop and Hayling part B tests), initiation (Hayling Part A), planning (Tower of Hanoi), and selective attention or mental flexibility (Trail Making test Part B), which are directly or indirectly related to inhibitory control (Moses et al., 2004).

#### 2.1.1Measurement of Inhibitory Control, and Initiation

To evaluate inhibitory control we applied the Stroop test (as marker of interference control) and Hayling test part A/B (as a marker of initiation or inhibition of a semantically automatic response). Stroop task is typically used as an index of inhibition in clinical and experimental studies. However, it has been criticized as a poor measure of genuine inhibition, therefore, in addition to: Stroop test, we used Hayling test.

In the Stroop test, participants were required to read aloud as fast and as accurately as possible (Reading), a list of 40 color names printed in black type. Then, they named aloud 40 colored patches (Naming). In the interference condition, they named aloud the colors in which the names of 40 other colors (Conflicting naming) were printed.

The Hayling test consists of two sections (A and B; Burgess & Shallice, 1996), each containing 14 sentences in which the final word is omitted (semantically constrained context). Respondents are required to listen and provide, as fast as possible, a word to complete each sentence. In section A (automatic generation, initiation), the provided word must be an appropriate one (e.g. the captain wanted to stay with the sinking… ship.). In section B (inhibition), participants are required to provide a word which makes no sense at all in the context (e.g. the captain wanted to stay with the sinking peanut). In the present study, we considered scores on Hayling Part A as indices of initiation performance, and those on Part B as indices of inhibition control (inhibition of unwanted responses).

#### 2.1.2 Measurement of Mental Flexibility and Selective Attention

The Trail Making test (part A and B) is a test of speed, and the examiner takes into account both the time taken and efficiency to carry out the test. Part A consists of encircled numbers from 1 to 25 randomly spread across a sheet of paper. Respondents are required, to connect the numbers in order, beginning with 1 and ending with 25, as fast as possible. Part B requires the respondents to connect numbers and letters in an alternating pattern (1-A-2-B-3-C, etc.) as fast as possible. In general, scores are calculated by adding the time taken by the respondents to complete both Parts. Because Part B requires more thought processing and attention on behalf of the respondents, we used only this part (Lezak, 1995). Participants were required to draw, as fast as possible, links between numbers and letters on a page in an alternating sequence.

#### 2.1.3 Measurement of Planning

The Tower of Hanoi task involves reaching a goal state through the execution of a series of moves. The objective is to move a pyramid of variously sized disks from one peg (start peg) to another (goal peg). Only one disk could be transferred per move and it had to be smaller than the disk underneath. Participants are required to accomplish the task with the minimum of moves (three-disk: 7 moves; four-disk: 15 moves). Each participant was shown the materials and told the rules. The experimenter then demonstrated how the problem could be resolved with 3 disks. The participants were told that they had to solve the same problem with 4 discs.

Performance indices for the Stroop test were calculated in terms of the interference means (Conflicting naming time-((Reading time X Naming time)/(Reading time + Naming time))) X - 1, with higher score corresponding to poorer inhibitory control. For the Tower of Hanoi, Trail Making, and Hayling tests performance indices were the times spent by participants to complete each test, measured with a stopwatch.

### 2.2 Assessment of Dispositional and Situational Aggressive Tendencies

To measure aggressive tendencies before experimental feedback (dispositional measure) we used standardized French versions of the Aggression Questionnaire (AQ; developed by Buss and Perry in 1992 and translated into French by Masse in 2001) in the first part of the study. The AQ contains four subscales including physical aggression (9 items), verbal aggression (8 items), anger (7 items), and hostility (5 items). Ratings of how the participant would usually respond are given on a 5-point intensity scale ranging from 1 (never) to 5 (always) depending on his/her habitual behavior.

For the second assessment of aggressive tendencies, in order to measure individuals’ situational variations in aggressive tendencies, we used a self-reporting method developed by O’Connor, Archer, & Wu (2001). The Aggressive Provocation Questionnaire or APQ is a widely used measure and is available in two formats: a 33 vignette and a 12 vignette versions. In the first study we used the 12-item French version (validated by Pahlavan, Amirrezvani, & O’Connor, 2010) of APQ. Following the APQ’s general instructions, the participants were asked to imagine being in a series of situations and to indicate (a) how s/he would feel in each situation (angry, frustrated, or irritated), measured on a 5-point intensity scale ranging from 0 (not at all) to 4 (extremely), and (2) how s/he would react to each situation by choosing one of five randomly-ordered action responses (avoidance, denial, distant anger, assertive behavior, or aggressive behavior). Following the O’Connor et al. procedure, the participants’ behavioral scores were calculated adding up the number of situations checked for each behavioral choice (only one choice out of 5 per situation) and dividing this sum by the total number of items (12 scenarios for each experimental session). Emotional scores were equal to the sum of the intensity value on each emotional dimension.

### 2.3 Procedure

After having read and signed the first part of an informed consent form and during a first session (20-30 minutes), participants completed a series of tests measuring executive capacities, and a questionnaire measuring aggressive tendencies (AQ). In the second experimental session (30-45 minutes), week after, participants performed the perceptual task inducing experimental condition before completion of the executive tests assessing the same cognitive capacities and filling out the questionnaire measuring aggressive tendencies (APQ). At the end of the second experimental session, participants read and signed the second part of the consent form. Then after, participants probed for suspicion and skepticism, but none reported knowledge of the study’s purpose. Finally, they were thoroughly debriefed and thanked.

### 2.4 Statistical Analysis

The missing data (about 3%) were replaced by participants’ average scores for each measure. The psychometrical qualities of the scales were examined. Cronbach’s alphas were calculated to assess the internal reliability of all the scales and subscales. Congruent validity was evaluated by correlating scores from different scales. The reliability (Cronbach’s alphas) was generally high (AQ: ranged from .89 to .97; APQ: ranged from .43 to .79), and inter-correlations between these scales were generally good and acceptable (anger AQ/APQ *r* = .43; AQphysical aggression/APQAggression *r* =.62). We also checked the distribution of our data and tested for variance heterogeneity. The data were normally distributed and analyses did not provide evidence that the assumption of homogeneity of variance had been violated, except for aggressive reactions, Bartlett *χ²* test: *F* 9 = 59.34, *p*<.000. Therefore, the behavioral scores were transformed into square roots.

## 3. Results

### 3.1 Relationship between Impaired Executive Performances and Aggressive Tendencies

As shown in Table 1, measures of aggressive tendencies (AQ) before inducing experimental conditions were significantly and positively correlated with chronically impaired performance on the executive function tests. More time was observed for more aggressive individuals. Note also that the same tendencies were found for aggressive tendencies (APQ) and performance on the executive tests after inducement of experimental conditions. Therefore our results are consistent with those reported in the studies published to date, and extend it to contextual measurements of aggressive tendencies and executive performance using a battery of neuropsychological tests. In sum, consistent with our correlative hypothesis, higher executive scores (higher performance on inhibition, planning, initiation and selective attention or mental flexibility) were observed for non-aggressive individuals before and after experimental manipulation.

**Table 1:** Observed inter-correlations between aggressive tendencies and executive functions scores before and after experimental feedbacks

AQ’s Subscales/Executive Functions	Physical Aggression	Hostility	Anger	Verbal Aggression				
Inhibition (Stroop)	.32	.16	.26	.15				
Planning (Tower of Hanoi)	.39	.40	.38	.20				
Initiation (HaylingA)	.50	.24	.44	.30				
Inhibition (HaylingB)	.56	.19	.47	.49				
Mental Flexibility (TMTB)	.54	.37	.48	.50				

APQ’s Sbscales/Executive Functions	Anger	Frustration	Irritation	Avoidance	Denial	Distant Anger	Assertive Responses	Aggressive Responses
Inhibition (Stroop)	.66	.59	.44	-.50	-.42	-.26	.19	.63
Planning (Tower of Hanoi)	.37	.32	.16	-.28	-.33	-.27	.13	.46
Initiation (HaylingA)	.44	.41	.35	-.58	-.50	-.20	.44	.40
Inhibition (HaylingB)	.62	.41	.62	-.68	-.49	-.18	.37	.56
Mental Flexibility (TMTB)	.61	.47	.53	-.58	-.47	-.23	.28	.61

Note: *r* >.24 are significant at p < .05

### 3.2 Effects of Experimental Feedbacks on Aggressive Tendencies and Executive Controls

As an exploratory step of our research, we analyzed separately the data from the first and the second experimental sessions, using a 2 × 3 factorial analysis (Gender x Feedback). A follow-up analysis was also conducted in order to compare the data from the first to the second session, with a 2 × 3 × 2 factorial analysis (Gender x Feedback x Experimental session), in which the latter factor was treated as repeated-measures.

Multivariate analysis of the scores on AQ scales and those on executive functions showed a significant effect involving gender of the participants, *F* (4, 51) =11.02, *p*<.000, Λ =0.52; *F*(4, 51) =2.39, *p*<.06; Λ =0.84.

Univariate analyses of these effects revealed that men were more angry, and physically, and verbally more aggressive than women (see Table 2). Univariate analyses for executive performance showed only a significant gender-related main effect for inhibition capacity (Hayling part B). As shown in table 2, compared to women, men seemed to have more difficulties to inhibit competing response. No other significant effect related to experimental design was found.

**Table 2:** Synthesis of Variance Analyses of the data from First and Second Experimental Sessions

First Experimental Session	Average Scores				*df*	*F*	*p*	*η*^2^
Main effects								
Aggressive tendencies (AQ):								
Gender	Women		Men					
Anger	15.17		21.77		1, 54	14.85	.000	.47
Aggression:								
Physical	13.33		25.23		1, 54	32.73	.000	.62
Verbal	10.40		15.23		1, 54	11.62	.002	.42
Executive Function:								
Gender								
Inhibition(Hayling part B)	197.63		204.50		1, 54	6.71	.02	.33

Second Experimental Session	Average Scores							
Main effects								
Measures of aggressive tendencies (APQ):								
Gender	Women		Men					
Anger	27.77		33.97		1, 54	31.09	.000	.60
Aggression	0.067		0.192		1, 54	10.85	.002	.10
Avoidance	0.244		0.150		1, 54	4.64	.04	.29

Feedback								
Conditions	Neutral	Failure		Success				
Anger	27.80	37.90		26.90	2, 54	40.23	.000	.66
Frustration	20.10	22.35		19.95	2, 54	11.92	.000	.43
Irritation	31.60	36.90		29.60	2, 54	14.41	.000	.46
Aggression	0.054	0.317		0.017	2, 54	15.43	.000	.52
Assertive reaction	0.375	0.529		0.179	2, 54	8.61	.001	.40
Avoidance	0.179	0.037		0.375	2, 54	19.92	.000	.58
Denial	0. 179	0.025		0.158	2, 54	10.35	.001	.46
Distant-anger	0.213	0.092		0.271	2, 54	10.32	.000	.40

Measures of Executive Functions:								
Feedback								
Conditions	Neutral	Failure		Success				
Inhibition:								
Stroop	1.28	3.41		0.48	2, 54	21.68	.000	.54
Hayling B	200.00	210.20		193.55	2, 54	13.43	.000	.34
Initiation:								
Hayling A	32.42	36.70		30.00	2, 54	6.97	.002	.45
Mentally flexible:								
TMTB	48.25	54.05		45.15	2, 54	10.68	.000	.41

Interaction effects of Gender and Feedback conditions:								
Measures of Aggressive tendencies:								
Frustration:					2, 54	4.19	.02	.27
Women	19.20	22.90		19.20				
Men	21.00	21.80		20.70				
Aggressive Reaction:					2, 54	3.45	.04	.24
Women	0.000	0.183		0.017				
Men	0.108	0.450		0.017				
Distant anger:					2, 54	8.19	.001	.36
Women	0.142	0.117		0.158				
Men	0.283	0.067		0.383				

Multivariate analysis of the scores on APQ scales after introducing experimental feedbacks showed significant main effects involving gender, *F*(7, 48) =7.67, *p*<.000, Λ=0.48, experimental feedback, *F*(14, 96) =10.15, *p*<.000; Λ=0.17, and a significant gender X experimental feedback interaction, *F*(14, 96) =3.41, *p*<.001; Λ=0.45.

As shown in table 2, univariate analyses of these effects revealed that the scores on anger, frustration, and irritation were highest under failure condition, but lowest under success condition. The participants’ aggressive, and assertive scores were also highest under failure condition, and lowest under success one. The tendencies reversed in the case of avoidance, denying, and distant-anger (see Table 2).

Univariate analyses showed also significant interaction between gender and feedbacks for the scores of frustration, as well as for aggressive and distant-anger as behavioral choices. Facing to the conflict situations, compared with men, women expressed more frustration under failure condition and less under other feedback conditions (see Table 2). Regarding behavioural choices, even though men generally chose more frequently aggressive responses, these differences were more noticeable under failure condition. At the same time, distant-anger was most frequently chosen by men and women under success condition. The main effect of gender was noticeable on the scores of anger, aggression, and avoidance. In general men’s strategies to cope with conflict situations seemed to be based on approach (anger and aggression), whereas women’s strategies were based on avoidance (frustration and avoidance). Follow-up analyses using the participants’ dispositional aggressive tendencies (scores on the AQ subscales) as covariable showed the same significant effects.

Multivariate analysis for executive function showed a significant main effect involving experimental condition, F (10, 100) =5.06, *p*<.000; Λ =0.44. As shown in table 3, univariate analyses of these effects revealed that under failure condition participants showed lowest, and under success condition highest executive performances for the capacities of inhibition, initiation, and being mentally flexible (see Table 2).

**Table 3:** Synthesis of variance analyses comparing the data of the first experimental session with those of the second one

Interaction effects: Experimental Session/Feedback	Average Scores			*df*	*F*	*p*	*η*^2^
	Neutral	Failure	Success
Measures of Aggressive tendencies:							
Anger:				2, 54	5.04	.01	.29
First	2.81	2.77	2.34				
Second	2.32	3.15	2.24				
Aggressive Reaction:				2, 54	5.81	.005	.31
First	0.341	0.278	0.171				
Second	0.0.096	0.457	0.058				

Measures of Executive Functions							
Inhibition (Stroop)				2, 54	14.93	.0001	.47
First	1.30	2.291	1.277				
Second	1.278	3.408	0.481				
Inhibition (Hyling B)				2, 54	41.73	.0001	.66
First	201.20	204.60	197.40				
Second	200.000	210.20	193.550				
Initiation (Hayling A)				2, 54	19.47	.0001	.52
First	32.450	34.900	32.500				
Second	32.400	36.700	30.000				
Planning (Tower of Hanoi)				2, 54	38.26	.0001	.65
First	35.35	34.30	34.35				
Second	33.30	35.90	30.55				
Mentally flexible (TMTB)				2, 54	69.86	.0001	.75
First	50.15	50.60	50.25				
Second	48.25	54.05	45.15				

### 3.3 Analyses Comparing the Data before and after Experimental Feedback

In order to evaluate the difference between two experimental sessions on aggressive tendencies, we compared average scores of the anger subscales of the AQ to those of the APQ. For aggressive responses, we analyzed frequency of aggressive responses on both scales. Because the scores on the physical aggression subscale of the AQ were highly correlated with the scores on aggressive responses on APQ, for former questionnaire, we took into account only responses corresponding to strong agreement (very often applies to me=1 and otherwise=0) on each item of this subscale. Then its average frequencies were calculated and transformed into square roots in order to be compared with APQ aggressive choices.

The data were examined with a 2 × 3 × 2 factorial analysis (Gender x Feedback x Experimental session), in which the latter factor was treated as repeated-measures.

Separate analyses of variance on anger and aggression scores indicated a significant interaction effect involving feedback condition and experimental session for both measures. As shown in Table 3, the participants were angrier and more aggressive after having received a negative feedback (see Table 3).

Analyses of the scores on different aspects of executive control revealed also a significant interaction effect involving experimental feedbacks and sessions. Participants’ performance on inhibition tests was worse after negative feedback and best after positive feedback, Stroop (see Table 3).

As shown in Table 3, after receiving negative feedback, the participants also had more difficulties, and took more time to initiate (Hayling Part A), to plan (Tower of Hanoi) their actions, and showed a relative deficit to concentrate their attention selectively (TMTB).

## 4. Discussion

Consistent with studies published to date, our first study provided evidence of a dispositional relationship between poor executive functioning and aggressive tendencies, and extended it to situational level. It showed that while a positive affective context resulted in a better executive performance and less aggressive tendencies, a negative feedback interfered significantly with participants’ performance and fostered aggressive feelings and actions. This fact may in part explain why individuals who grow in adverse environmental might develop poor executive capacities, and be emotionally vulnerable and more akin to aggression and antisocial behavior. As mentioned by Kochanska (Kochanska et al., 1997), development of executive controls and self-regulations is a crucial step of socialization. However, in spite of an early-development of these capacities, our data showed that situational characteristics could alter individuals’ sensibility and control capacities in adulthood. Adults are supposed to be able to regulate their affective experiences and shift their attention away from the negative cues related to anger or other impacts of immediate self-threatening environment. Nevertheless, our data clearly showed that regardless of age or developmental step of individual, self-threatening context could cause a deficit in social information processing, which result in a high emotional reactivity. To our knowledge, this is the first time that executive control and impulsivity (or any other measure of reactive control) have been shown to have direct relations to dispositional as well as situational aggressive tendencies in adults.

However, the results of our first study established that relations between executive controls and behavioral adjustment were not due solely to the individual dispositional in a sample of students, as the most convenient population. Yet, given the nature of our research and our objectives, further studies with other populations, specifically in different psychiatric fields, would be of great interest. In this vein, we realized our second study in order to extend our finding to two other populations.

## B. Second Study

Principal objective of our second study was to reassess and extend the results of our first study. Therefore, its’ primary aim was to examine the relation between executive control and aggressive tendencies using a feedback paradigm in three different samples. Based on prior findings and the results of our first study, for our student sample we predicated the same correlative relation (positive correlations) between executive deficits and aggressive tendencies before and after inducing experimental feedbacks (Correlative Hypothesis), and causal effect of feedback on those relations (Causal Hypothesis). We also examined our gender-related hypothesis related to aggressive tendencies.

However, as specific hypothesis, for participants with dysexecutive syndroms we hypothesized that causal effect of experimental feedback would become stronger after receiving a negative feedback. That is, for participants with dysexecutive syndroms we predicted an additive effect of executive deficits and behavioural problems due to negative evaluation of their performance. Our specific hypothesis regarding health care workers predicted opposite tendencies. Given their professional duties and expertise, we hypothesized that their behavior may be less, chronically and contextually, influenced by impulsive processes induced experimental feedbacks.

## 1. Method

The results of the first study has supported our preparatory work and provided convincing evidence that the work has been thoroughly thought through. However, few methodological problems subsisted regarding adaptation of the experimental material and procedure as well as standardization of the assessment of executive capacities for our three samples and through experimental phases. Therefore, for our second study we used a digitalized version of some of the tests. In addition, for having a pure measure of selective attention and inhibition control, we replaced Hyling A/B and Stroop tests by a pure task of reaction time (Go-No/go) and Modified Card Sorting Test (WCST; Milner, 1964). For measuring aggressive tendencies before and after inducing experimental feedbacks, we decided to use a long version of the same self-reporting questionnaire (APQ; Pahlavan et al., 2007). We added also a check experimental feedbacks procedure. Otherwise, the procedure represented a replication of the first study, and used the same material.

### 1.1 Participants

#### 1.1.1 Non-Clinical Sample

*Student sample:* Sixty students of psychology (female=30, male=30) aged between 19 and 28 years (*M* = 21.91 years, *SD* = 3.50) from University Paris-Descartes participated in a two-session study. They were recruited via announcement put up around Faculty. Participants were all right-handed native French speakers, and none reported any neuropsychological or developmental disorder.

*Health care workers:* Forty-five health care workers (female=30, male=15) aged between 22 and 57 years (*M* = 38.62 years, *SD* = 11.39) from Delafontaine Hospital (Pole Casanova) participated in a two-session study. They were recruited via announcement put up around Hospital. They were all right-handed French speakers, and none reported any neuropsychological or developmental disorder.

All subjects from non-clinical samples participated on a voluntary basis, and after having read and signed the same double informed consent form used for our first study before starting and after finishing the experimental sessions. Participants were randomly assigned only to one level of the experimental condition.

#### 1.1.2 Clinical Population

For our clinical sample, twenty-four right-handed native French-speakers (female = 12, male = 12 and) aged between 22 to 57 years (*M*= 38.62 years, *SD*= 15.50 years) from Delafontaine Hospital (Pole Casanova) with dysexecutive syndromes were recruited. They were recruited via word-of-mouth from Delafontaine Hospital (Pole Casanova) by research assistant (one of the coauthor of the present paper). There were no specific criteria for selection other than to find adults with clinically mild or moderate relapsing-remitting dysexecutive syndromes. Their dysexecutive syndromes were diagnosed by a physician from Delafontaine Hospital, through every day application of the traditional tests (Alderman, Burgess, Emslie, Evans, & Wilson, 2003) and were due to frontal lesion (17: 9 Female and 8 males), multiple sclerosis (4: 3 Female and 1 male) or brain trauma (3 males) less than 4 years duration (M=2.5 years, SD =1.50). The study was approved by an institutional ethical board from Delafontaine Hospital and all participants agreed to a consent form in accordance with the ethical principles of the American Psychological Association.

### 1.2 Experimental Condition

The procedure for inducing experimental feedbacks represented a replication of the first study, and used the same material. The experimenter presented the white-spotted page for 5 seconds and then introduced the experimental feedback (failure, success, and neutral) by means of three different messages, apparently based on the participant’s estimation. At the end of the second experimental session, induction of experimental feedbacks was checked using a check experimental procedure.

## 2. Material and Measures

### 2.1 Measures of Executive Functions

We mostly measured the same executive capacities using the digital version of the same tests (planning: Tower of Hanoi, selective attention: Trail Making test Part B), except for inhibition capacity. For assessment of inhibition controls we used two other neuropsychological tests: Modified Card Sorting Test (WCST; Milner, 1964) and Go/No-go tasks.

The Wisconsin Card Sorting Test (WCST) is a neuropsychological test of categorization, measuring the ability to display flexibility whenever rules of categorization changes. Initially, a number of stimulus cards are presented to the participant. For digital version, 4 stimulus cards were presented on the screen all the time. For each trial one to-be-matched card was presented and participant had to touch one of the 4-stimulus cards as being matched (because of the shape, color, or number of shape on the to-be-matched card) with the trial card. In the case of the non-clinical population or a sample of persons with slight or moderate DES the flexibility measure is not useful, because the number of accomplished categories is always maximal. Therefore, we analyzed only inhibitory capacity or perseveration errors measured during this task in terms of time spent by the participant to complete the task (categories achieved) with false response in spite of the rule (perseverative errors; Berg, 1948; Monchi, Petrides, Petre, Worsley, & Dagher, 2001).

Go/no-go test refers to a pass/fail test (or check) principle. In psychology Go/No-go tests are used to measure a participant’s capacity for sustained attention and response control. In the case of our second study, during Go test participants were required to press a button each time a centered black circle of 2 cm diameter was presented on the screen (given stimuli: press a button – Go) for a number of trials. During No-go, the participants were asked to inhibit that action under the same condition (not press that same button when the black circle was presented-No-Go). Scoring was based on the time spent to accomplish trials under each condition. GO condition score was considered as a measure of selective attention, and No-go condition score as inhibitory capacity.

The order of tests and tasks in both sessions was as follow: each session started with The Wisconsin Card Sorting Test and Tower of Hanoi tasks followed by Go/No-go task and The Trail Making test part B.

The presentation of a digitized version of the tests, collection of responses, and latency of the responses were controlled and recorded by an IBM compatible computer.

### 2.2 Measures of Dispositional and Situational Aggressive Tendencies

Given conceptual differences between AQ and APQ, in the second study we used a long version of APQ (APQ, Pahlavan et al., 2007), 12 items of which were used in the first and 12 others in the second part of the study. For both sessions, the APQ was applied following its’ general instructions, the participants were asked to imagine being in a series of situations and to indicate (a) on 5-point scales how s/he would feel in each situation (angry, frustrated, or irritated), and (2) how s/he would react to each situation by choosing one of five randomly-ordered action responses (avoidance, denial, distant anger, assertive behavior, or aggressive behavior).

### 2.3 Procedure

After having read and signed the first part of an informed consent form and during a first session (20-30 minutes), participants completed a series of tests measuring executive capacities, and a questionnaire measuring aggressive tendencies (12-item APQ). In the second experimental session (30-45 minutes), week after, participants performed the perceptual task inducing experimental condition before completion of the executive tests and filling out the questionnaire measuring aggressive tendencies (12-item APQ). At the end of the second experimental session, participants were asked to indicate on two 7-point intensity scales ranging from 0 (not at all) to 7 (extremely) the extent to which they anticipated to complete the experimental task with failure and success (Success-Failure scales). Then after, participants probed for suspicion and skepticism, but none reported knowledge of the study’s purpose. Finally, they signed the second part of an informed consent form before being thoroughly debriefed and thanked.

### 2.4 Statistical Analyses

After replacing the missing data (about 3-5%) by participants’ average scores for each measure, the psychometric qualities of the APQ’s affective scales were evaluated. The psychometric qualities of the APQ’s affective scales were highs (Cronbach’s Alphas: Anger=0.85, Frustration=0.88, Irritation=0.88) and their inter-correlations acceptable (Anger- Frustration =0.51, Anger-Irritation=0.51, Irritation-Frustration=0.51). We also checked the distribution of the data and tested for variance heterogeneity. Except for aggressive reactions, Bartlett *χ²* test: *F* 9 = 30.53, *p*<.00, and distant-anger, Bartlett *χ²* test: *F* 9 = 12.92, *p*<.01, the data were normally distributed. Therefore, the behavioral scores were transformed into square roots.

## 3. Results

### 3.1 Experimental Feedbacks Check

Before testing our research hypotheses, participants’ scores on Success-Failure scales were analyzed in order to verify the effect of experimental feedbacks. A 2 × 3 × 3 × 2 (Gender × Sample type X Experimental Feedback X Scale type) ANOVA was used in which the latter factor was treated as a repeated measure. Analysis of ratings revealed significant Experimental condition x Scale type interaction, *F* 2, 110 = 3.84, *p* < .03, η²= 0.19. Follow up analyses showed significant effect of experimental conditions on failure-scale, *F* 2, 110 = 3.64, *p* < .03, η²= 0.18, with the highest failure expectation score for participants in success condition and the lowest failure expectation for participants in neutral condition (*M*= 4.43 vs *M*= 4.12 vs *M*= 3.47). Reverse but not significant pattern was observed on the success expectation scores, *F* 2, 110 = 2.74, *p* < .06, η²= 0.16, *M*= 3.63 vs *M*= 3.69 vs *M*= 4.38. Thus, experimental manipulation produced groups that differed substantially in their average failure expectation.

### 3.2 Relationship between Impaired Executive Performances and Aggressive Tendencies

#### 3.2.1 Non-Clinical Sample

3.2.1.1 Students Sample

As shown in Table 4, for the students sample the measures of aggressive tendencies were positively correlated with impaired performance on the executive function tests, specifically after experimental feedbacks. More time was observed for more aggressive individuals. Therefore our results for students sample are consistent with those reported in existing literature, and confirm those we found in the case of our first study. In sum, consistent with our correlational hypothesis, higher executive scores (higher performance on perseveration, planning, attention, inhibition, and mental flexibility) were observed for non-aggressive individuals before and after experimental manipulation.

**Table 4:** Inter-correlations between the Executive Functions and the APQ’ subscales scores for Student sample (n=60)

	Anger	Frustration	Irritation	Avoidance	Denial	Distant Anger	Assertive Response	Aggressive Response
Before Experimental Feedback								
Perseveration (WCST) t1	.17	.18	-.01	-.13	-.17	.07	.05	.16
Planning (Tower of Hanoi) t1	.22	.24	.02	.04	-.16	-.00	.03	.09
Attention (Go) t1	.27	.23	.04	-.15	-.29	-.03	.31	.02
Inhibition (No-go) t1	.19	.20	.09	.00	-.25	-.04	.30	-.12
Flexibility (TMTB) t1	.25	.25	.10	-.00	-.16	-.09	.22	-.08

After Experimental Feedback								
Perseveration (WCST) t2	.21	.26	.26	-.03	-.36	-.15	.17	.29
Planning (Tower of Hanoi) t2	.14	.24	.17	.07	-.18	-.01	-.02	.14
Attention (Go) t2	.17	.25	.07	.01	-.09	.08	.13	-.10
Inhibition (No-go) t2	.14	.38	.18	-.19	-.27	-.02	.18	.24
Flexibility (TMTB) t2	.25	.28	.24	-.06	-.29	-.13	.20	.21

Note: *r* >.25 are significant at p < .05

3.2.1.2 Health Care Workers Sample

For the health care workers sample the major part of the correlations between the scores on the APQ subscales and impaired performance on the executive function tests were rather negative and relatively small, except those found between inhibition, on one hand with anger and on the other hand, with frustration before receiving experimental feedbacks. Thus, although the same tendencies were found between measures of affect ratings (anger, frustration, and irritation) and performance on the executive tests before and after experimental manipulation (see table 5), health care workers seemed to be able to manage their behavioral in terms of aggressive tendencies. In sum, consistent with our hypothesis, the measures of aggressive behavior were negatively correlated with the executive function scores, before and after experimental feedbacks (see table 5).

**Table 5:** Inter-correlations between the Executive Functions and the APQ’ subscales scores for Health Care Workers sample (n=45)

	Anger	Frustration	Irritation	Avoidance	Denial		Distant Anger	Assertive Response	Aggressive Response
Before Experimental Feedback									
Perseveration (WCST) t1	.24	.15	.02	.10		-.30	.05	.25	-.20
Planning (Tower of Hanoi) t1	.04	-.01	-.07	.32		-.29	-.08	.12	-.17
Attention (Go) t1	.25	.19	.10	.14		-.19	-.17	.19	-.13
Inhibition (No-go) t1	.26	.32	.09	-.02		-.20	-.21	.25	.00
Flexibility (TMTB) t1	.08	.02	.12	.10		-.06	-.23	.16	-.13

After Experimental Feedback									
Perseveration (WCST) t2	.15	.24	.09	.29		.31	.33	-.37	-.22
Planning (Tower of Hanoi) t2	-.11	.11	-.11	.26		.49	.06	-.35	-.13
Attention (Go) t2	-.11	-.02	-.18	.48		.33	.17	-.40	-.24
Inhibition (No-go) t2	-.00	.17	.04	.64		.28	.19	-.46	-.26
Flexibility (TMTB) t2	-.15	-.14	-.16	.41		.27	-.05	-.19	-.20

Note: *r* >.32 are significant at p < .05

#### 3.2.2 Clinical Sample (DES)

As shown in Table 6, for the persons with dysfunctional executive syndromes correlations between the measures of aggressive tendencies (negative affective states and behavioral choices) and impaired executive performances before experimental feedbacks were rather negatives or smalls, except for planning and flexibility (see table 6). The measures of anger and frustration as well as those related to aggressive behavior were positively and significantly correlated with impaired performance on the planning, and flexibility scores, specifically before experimental feedbacks. Most interesting, the measure of aggressive behavior was negatively and significantly correlated with impaired performance on the perseveration score before experimental feedbacks. However, after receiving experimental feedbacks correlation between those measures became highly positive (before: *r*=-.33; after: *r*=.38). Therefore our results for the persons with DES are partly consistent with our hypothesis, higher executive scores (better performance on perseveration, attention, and flexibility) were observed for non-aggressive persons with DES specifically after experimental manipulation. However, given small sample size (n=24) these results must be considered with some reserves.

**Table 6:** Inter-correlations between the Executive Functions and the APQ’ subscales scores for Clinical-DES sample (n=24)

	Anger	Frustration	Irritation	Avoidance	Denial	Distant Anger	Assertive Response	Aggressive Response
Before Experimental Feedback								
Perseveration (WCST) t1	.07	.15	-.06	.05	.02	.24	.13	-.34
Planning (Tower of Hanoi) t1	.32	.24	-.01	-.20	.03	.17	-.14	.15
Attention (Go) t1	-.13	-.01	.03	-.34	.17	.18	.06	.11
Inhibition (No-go) t1	-.27	-.10	-.02	-.11	.23	.13	.00	-.02
Flexibility (TMTB) t1	.33	.28	.07	-.12	-.06	.04	-.09	.21

After Experimental Feedback								
Perseveration (WCST) t2	.17	.13	.17	-.37	-.00	.10	-.05	.38
Planning (Tower of Hanoi) t2	.19	.17	.13	.21	-.20	.31	-.16	-.10
Attention (Go) t2	-.08	.01	-.01	.12	.16	.32	-.41	-.00
Inhibition (No-go) t2	-.18	-.07	-.07	-.05	.11	.23	-.39	.23
Flexibility (TMTB) t2	.22	.15	.17	-.07	-.23	.35	-.15	.18

Note: *r* >.35 are significant at p < .05

### 3.3 Effects of Experimental Feedbacks on Aggressive Tendencies and Executive Controls: Comparing the Data before and after Experimental Feedback

In order to verify our causal hypotheses, we analyzed and compared directly the data from the first to the second experimental sessions for all participants. To evaluate the difference between two experimental sessions, we conducted separate analyses of variance on affective scores on the APQ’s subscales (anger, frustration, irritation) and compared average scores of the behavioral responses. For verifying the difference between two experimental sessions on executive performances, we used a 2 × 3 × 2 factorial analysis (Gender x Feedback x Experimental session), in which the latter factor was treated as repeated-measures. In addition, we conducted separate analyses for each executive performance.

#### 3.3.1 Non-Clinical Sample

3.3.1.1 Students Sample

• *Analyses of Aggressive Tendencies*

Analyses of variance on affective scores on the APQ’s subscales (anger, frustration, irritation) indicated significant main effects of experimental sessions and feedback conditions only for frustration scores. In general, compared to the first session, the participants significantly expressed more frustration during the second session. In addition, they seemed to be more frustrated after having received a negative feedback than a positive or neutral one (see Table 7).

**Table 7:** Synthesis of Variance Analyses Comparing the data of the First Experimental Session with those of the Second Experimental Session for Students Sample

Score on APQ		Average Scores									*df*	*F*	*p*	*η*^2^
Main effects														
Feedback														
Conditions		Neutral			Failure			Success						
Frustration		23.72			21.15			15.30			2, 54	5.08	.01	.30
Aggression		0.77			0.16			0.16			2, 54	3.41	.05	.24
Denial		0.12			0.20			0.12			2, 54	3.37	.05	.24
Experimental Session		First Session					Second Session							
Frustration		18.77					21.35				1, 54	15.17	.001	.47
Aggression		0.12					0.14				1, 54	5.85	.05	.31
Affirmation		0.51					0.40				1, 54	24.13	.001	.47
Anger-distant		0.09					0.12				1, 54	6.03	.05	.32
Avoidance		0.12					0.19				1, 54	15.23	.001	.47

Interaction:														
Experimental Sessions X		First Session					Second Session							
Feedback conditions		Success	Failure			Neutral	Success	Failure		Neutral				
Aggression		0.11	0.07			0.17	0.04	0.23		0.17	2, 54	15.43	.001	.56
Denial		0.17	0.17			0.13	0.24	0.07		0.12	2, 54	10.35	.001	.39
Anger-distant		0.08	0.08			0.10	0.16	0.10		0.09	2, 54	3.43	.04	.23
Experimental Sessions X		First Session					Second Session							
Gender		Male		Female			Male		Female					
Denial		0.17		0.15			0.11		0.17		1, 54	4.58	.05	.25
Experimental Sessions X		First Session					Second Session							
Feedback conditions X		Success	Failure			Neutral	Success	Failure		Neutral				
Denial											2, 54	4.25	.05	.27
Gender	Male	0.08	0.07			0.09	0.23	0.08		0.07				
	Female	0.08	0.10			0.10	0.10	0.13		0.13				

Executive Performances		Average Scores									*df*	*F*	*p*	*η*^2^
*Main effects*:														
Feedback conditions		Success				Failure		Neutral						
Selective Attention		81.30				91.25		82.90			2, 54	3.37	.05	.23
Experimental Sessions		First Session					Second Session							
Mental Flexibility		62.24					56.66				1, 54	7.07	.02	.34
Planning		72.51					58.00				1, 54	7.08	.02	.34

*Interaction Effects:*														
Experimental Sessions X														
Feedback conditions		First Session					Second Session							
		Success	Failure			Neutral	Success	Failure		Neutral				
Selective Attention		82.82	86.45			84.38	79.80	96.05		81.40	2, 54	6.60	.01	.33
Inhibition		108.69	97.28			105.87	101.49	124.96		97.92	2, 54	22.07	.001	.54
Perseveration		155.73	151.80			162.26	134.91	179.68		144.62	2, 54	11.23	.001	.42
Planning		81.18	63.43			73.16	52.80	73.26		47.93	2, 54	4.98	.05	.29
Mental Flexibility		71.40	59.38			55.94	49.77	74.52		74.52	2, 54	19.93	.001	.42
Feedback conditions X		Success				Failure		Neutral						
Inhibition											2, 54	5.09	.01	.40
Gender	Male	105.30				90.72		108.65						
	Female	104.88				122.53		95.13						
Feedback conditions X		First Session					Second Session							
Experimental Sessions		Success	Failure			Neutral	Success	Failure		Neutral				
Inhibition											2, 54	4.29	.05	.27
Gender	Male	109.94	94.63			113.59	100.71	104.82		103.73				
	Female	107.49	90.94			98.16	102.27	145.12		92.11				
Mental Flexibility											2, 54	3.60	.05	.25
Gender	Male	88.36	63.39			55.59	55.04	80.40		47.76				
	Female	54.44	55.38			56.30	44.50	68.63		43.65				

Note: Measurement unites for Executive performances are expressed in Millisecond

As shown in Table 7, analyses of behavioral choices showed a significant third order interaction between experimental feedbacks, experimental sessions, and gender only on distant-anger responses, and three significant second order interactions involving experimental feedbacks and experimental sessions on aggressive, distant-anger and denial responses.

Inspection of the data for third order interaction showed that the most frequent distant-anger responses for men were found in the success condition during second session. In the failure condition tendencies were the same, however reversed tendencies were found under the neutral condition. For women the frequencies of distant-anger responses increased from first session to second one, independent on the modality of the feedbacks. These tendencies were supported by a second order interaction involving experimental feedbacks and experimental sessions.

Second order significant interactions of experimental session and experimental feedbacks on aggressive and denial responses revealed that although participants chose more frequently denial under the success condition et aggression in the failure condition, these tendencies were more noticeable during the second session than the first one.

ANOVAs of data related to behavioral choices revealed also significant simple effect of experimental session for all choices except for denial ones. In general, compared to first session, the participants chose more frequently aggressive, distant-anger, and avoidance responses during second session. Reverse tendencies were found for assertive choices.

• *Analyses of Executive Function Performance before and after Experimental Feedbacks*

In order to evaluate the difference between two experimental sessions on executive performances, we used a 2 × 3 × 2 factorial analysis (Gender x Feedback x Experimental session), in which the latter factor was treated as repeated-measures.

Analyses of variance revealed significant interaction effect involving experimental session and feedback condition for each measured executive capacity, including perseveration, planning, attention, inhibition, and mental flexibility. In all cases, under the success condition participants’ performance became better from the first session to the second one, involving shorter executive times for second session than those observed during the first session. Reverse tendencies were observed for those in the failure condition (see Table 7).

As shown in Table 7, analyses of inhibition capacities revealed a significant third order interaction between experimental feedbacks, experimental sessions, and gender, supported by a second order interaction between gender and experimental feedback. Inspection of data for third order interaction showed the shortest and the longest time decision for women under the failure condition respectively before and after having received experimental feedbacks.

The same significant third order interaction between experimental feedbacks, experimental sessions, and gender for mental flexibility was found, with an increased in time decisions for women as well as for men under the failure condition after receiving experimental feedback, reverse tendencies were observed in success and neutral conditions for women as well as for men.

3.3.1.2 Health Care Workers Sample

• *Analyses of Aggressive Tendencies*

Separate analyses of variance of affective scores on the APQ’s subscales (anger, frustration, irritation) indicated significant main effects of experimental sessions for frustration scores. In general, compared to the first session, the participants significantly expressed more frustration during the second session (see Table 8).

**Table 8:** Synthesis of Variance Analyses Comparing the data of the First Experimental Session with those of the Second Experimental Session for Health Care Workers

Score on APQ		Average Scores						*df*	*F*	*p*	*η*^2^
*Main effects*											
Experimental Session		First Session			Second Session						
Frustration		18.60			21.81			1, 54	9.47	.01	.44
Affirmation		0.48			0.55			1, 54	4.50	.05	.32

Executive Performances		Average Scores						*df*	*F*	*p*	*η*^2^
*Main effects*:											
Experimental Sessions X		First Session			Second Session						
Perseveration		192.41			173.81			1, 39	6.82	.02	.38
Planning		125.95			71.58			1, 39	50.29	.001	.75
Selective Attention		96.36			138.31			1, 39	29.95	.001	.66
Inhibition		93.45			115.93			1, 39	24.75	.001	.63
Mental Flexibility		92.81			67.86			1, 39	6.52	.05	.38

*Interaction Effects*:											
Experimental Sessions X		First Session			Second Session						
Feedback conditions		Success	Failure	Neutral	Success	Failure	Neutral				
Planning		147.27	126.43	75.29	69.37	69.37	104.15	2, 39	3.51	.05	.29
Feedback conditions X											
Planning		Success		Failure		Neutral		2, 39	3.51	.04	.29
Gender	Male	127.23		68.89		79.96					
	Female	89.43		12703		99.99					
Experimental Sessions X		First Session			Second Session						
Feedback conditions		Success	Failure	Neutral	Success	Failure	Neutral				
Planning								2, 39	3.85	.05	.30
Gender	Male	183.28	88.85	94.47	71.17	49.10	65.65				
	Female	111.27	164.01	113.83	111.27	90.06	86.14				

Note: Measurement unites for Executive performances are expressed in millisecond

As shown in Table 8, analyses of behavioral choices showed a significant main effect of experimental session for assertive behavior choices. Compared to the first experimental session, in the second session participants chose less frequently assertive responses to cope with the APQ’S conflict situation.

• *Analyses of Executive Function Performance before and after Experimental Feedbacks*

Analyses of the variance related to health care workers’ executive performances revealed a significant main effect of experimental sessions for all executive capacities. As shown in table 8, participants took more time to perform executive tasks related to perseveration, planning, and mental flexibility capacities, during the first experimental session than second one. Reverse tendencies were found when they performed the executive tasks measuring selective attention and inhibition, with more time observed during the second experimental session.

In addition, analyses of planning capacities revealed a significant third order interaction between experimental feedbacks, experimental sessions, and gender, supported by two second order interaction between gender and experimental feedbacks, on one hand and on the other hand between experimental feedbacks and experimental sessions. Inspection of the data showed that although the longest planning time decisions were observed for men under the success condition and for women under the failure condition, and that time decisions were shorter during the second experimental session than the first one, the decrease of time decision was the most noticeable after men received the experimental feedback (see Table 8).

#### 3.3.2 Clinical Sample

• *Analyses of Aggressive Tendencies*

Analyses of variance of the scores on the APQ’s subscales (affective scores and behavioral choices) indicated significant second order interactions between experimental sessions and feedback conditions only for aggressive, and avoidance behaviorals. In general participants in the failure condition chose more and those under the success condition chose less aggressive responses to cope with the APQ’s conflict situations after having received experimental feedbacks. As shown in Table 9, tendencies were reversed for avoidance choices. In fact, participants in the failure condition chose less and those under the success condition chose more avoidance as behavioral responses when facing with the APQ’s conflict situations after receiving experimental feedbacks.

**Table 9:** Synthesis of Variance Analyses Comparing the data of the First Experimental Session with those of the Second Experimental Session for Persons with DES

Score on APQ		Average Scores												*df*	*F*	*p*	*η*^2^
*Main effects*																	
Feedback conditions		Success				Failure				Neutral							
Aggression		0.20				0.29				0.08				2, 18	3.78	.04	.42
Experimental Sessions X		First Session						Second Session									
Feedback conditions		Success	Failure		Neutral			Success			Failure		Neutral				
Aggression		0.29	0.21		0.09			0.10			0.37		0.07	2, 18	16.16	.001	.69
Avoidance		0.21	0.32		0.21			0.36			0.12		0.28	2, 18	8.50	.01	.57

Executive Performances		Average Scores												*df*	*F*	*p*	*η*^2^
*Main effects*:																	
Feedback conditions		Success			Failure						Neutral						
Mental Flexibility		251.51			166.74						142.12			2, 18	9.28	.01	.58
Experimental Sessions		First Session							Second Session								
Selective Attention		139.61							306.37					1, 18	64.52	.001	.88
Inhibition		141.51							283.85					1, 18	63.72	.001	.88

*Interaction Effects*																	
Experimental Sessions X		First Session							Second Session								
Feedback conditions		Success		Failure			Neutral		Success			Failure	Neutral				
Planning		372.25		196.91			197.35		311.53			290.49	193.50	2, 18	5.36	.02	.48
Selective Attention		131.37		130.74			156.73		397.03			235.02	286.01	2, 18	5.79	.02	.49
Mental Flexibility		292.32		142.99			145.78		211.73			190.48	138.46	2, 18	10.31	.001	.60
Feedback conditions X		Success					Failure					Neutral					
Mental Flexibility														2, 18	3.85	.05	.42
Gender	Male	215.94					153.17					178.62					
	Female	287.11					180.30					105.62					

Note: Measurement unites for Executive performances are expressed in millisecond

• *Analyses of Executive Function Performance before and after Experimental Feedbacks*

Analyses of the executive performances revealed main significant effect of experimental session for inhibition, and selective attention. In all these cases, compared to the first experimental session, time decisions during second session were longer. Significant main effects of the experimental feedbacks on mental flexibility capacitie were found, too. The results showed the longest and the shortest flexibility time decisions respectively under the success and the neutral conditions.

In addition, analyses for executive performances showed two second order interactions involving experimental sessions and experimental feedbacks on planning and selective attention, on one hand and on the other hand, experimental feedbacks and gender for mental flexibility.

In the case of interaction between experimental feedbacks and experimental session on planning and selective attention capacities, under the failure condition participants’ performance became worse from the first session to the second one, involving longer executive times for second session than those observed during the first session. Under the success condition, reverse tendencies were observed; except for selective attention with the longest time decisions during second session (see Table 9).

As shown in Table 9, the configuration of the data for interaction between gender and experimental feedbacks on mental flexibility showed that although all participants under the success condition took more time for making a decision, women were the slowest.

## 4. Discussion and Conclusion

The results of these studies are informative regarding important issues. Consistent with existing literature, we found evidence of a positive dispositional relationship between poor executive functioning and aggressive tendencies, specifically for our student and clinical samples. Some individuals’ behaviour seem to be chronically influenced by impulsive processes the way participants’ behavior were influenced in the first assessment of the present research, independent of any experimental manipulations. Second, our results showed the same relation, in terms of situational or contextual control resource after our participants received experimental feedbacks. Thus, the relations of different executive processes and behavioral adjustment at the second assessment were not due solely to the individual dispositional differences in the variables. As predicted, it appears that increases in impulsiveness (negative emotionality and aggressive choices) due to negative feedback were concomitant with an inability to focus individuals’ attention on ongoing tasks (such as decreased performance in Trait Making Test, Tower of Hanoi, Go task), and thus were related to diminished inhibition control of task-irrelevant information (such as decreased performance in Stroop, WCST, No-go task). For our clinical sample, for example, we observed a concomitant increase of aggressive choices and decrease of executive capacities such as planning, attention, and flexibility, after receiving a negative feedback. More interesting, our results related to aggressive tendencies and executive performances in health care workers confirm the important role of the practice and training in efficient behavioral adjustment and regulation.

Regarding gender-related hypothesis, in spite of a general tendencies corresponding to more aggressive behavioral intention and higher emotional negativity in men than women, as predicted we found more emotional negativity in women and more aggressive behavioral intention in men after receiving a negative feedback. Unique gender-related difference in cognitive ability observed in the case of the first study was related to the inhibition, with women scoring higher than men on the inhibition section of the Hayling test, but more sensitive to the context of those adjustments (No-Go task). As mentioned in material section, the Hayling test produces a semantically constrained context, and these differences might be at least partially accounted for by women’s greater verbal ability. However, it is important to note that these executive differences disappeared after participants received a feedback and even reversed. This confirms the specificity relative to the effects of our experimental feedbacks on executive processes, and is a part of elements that were addressed in the present research.

However, the extent of these differences and their relation to the adjustment behavior remain to be further understood, and merit further investigations. More research is needed for testing these differences taking into account other emotional and executive characteristics of individuals. The findings of the current research suggest that different aspects of executive control are likely to play an important role in self-regulation across different contexts. As expected, our studies indicate that increases in impulsiveness due to negative feedback might be concomitant with an inability to voluntarily turn one’s attention away from the adverse characteristics of possible conflict, and thus might be related to diminished inhibition control of task-irrelevant information. Moreover, these relations seemed to be contextually moderated by anger. That is proneness to anger heightens individuals’ vulnerability to behavioral problems, depending on contextual characteristics. Therefore, it was assumed that anger contextually moderates relations of executive controls (voluntarily or reactive controls) to behavioral problems. Yet, our findings suggest that the contributions of self-regulatory processes such as executive controls to adjustment are more multifaceted and complex than have been acknowledged.

In summary, our finding suggest that executive control considered as inhibition of unwanted thought, initiation/planning new actions, and being selectively attentive, are jointly implicated in regulation of emotional behaviors such as aggression. In addition, the affective context influenced the level of these executive controls. While a positive affective context resulted in a better executive performance and less aggressive tendencies, a negative feedback interfered significantly with participants’ performance and fostered aggressive feelings and actions. This fact may in part explain why individuals who grow in adverse environmental might develop poor executive capacities, and be emotionally vulnerable and more akin to aggression and antisocial behavior. Yet, even though this programming might be in place very early in development, our data showed that situational characteristics could alter individuals’ sensibility and control capacities in adulthood.

It seems whatever the individual’s age or his/her developmental step, self-threatening context may cause a deficit in social information processing, which result in a high emotional reactivity. Thus, even though adults are supposed to control their affective experiences and shift their attention away from the negative cues related to anger, impacts of immediate self-threatening environment could alter their regulatory capacities. To our knowledge, this is the first time that executive control and impulsivity (or any other measure of reactive control) have been shown to have direct relations to dispositional as well as situational aggressive tendencies in clinical and non-clinical adults.

However, our research present some limits. Understanding the complex interaction effects between dispositional and situational differences in executive control of affective behaviors needs more appropriate statistical analyses, such as structural equation modeling (SEM). Our sample-size, specifically for our clinical sample, did not allow applying those analyses. Other methodological questions subsist regarding so-called “task purity” (Rabbitt, 1997), and to decisional processes involved in treatment of the text-evoked imagery. In fact, tasks available to assess executive function are “impure” in the sense that they may also make demands on a variety of other cognitive skills or functions that are supported by brain structures independent of frontal cortex (automatic control, Rabbitt, 1997). Our material lacks a pure measurement of working memory capacities. As a matter of fact, individuals experiencing negative emotional state, beset with self-doubts, might turn their attention inward and become self-preoccupied and devoted to emotional repair (searching a positive personal souvenir). In addition, conflict tasks (e.g. Stroop, No-go) require conflicting alternatives to be hold in mind (Moses & Carlson, 2004). Therefore, under the failure condition the working memory demands should be greater than those under the success condition, and envisioning failure scenarios easier (Wyer, 2004). If this analysis is correct, we would expect more anger and more aggressive tendencies whenever the working memory demands increase.

Our findings seemed also underscore the importance of decisional processes involved in the treatment of the text-evoked imagery. Even though, we focused on the intensity and frequency of a few discrete emotional experiences, and explored differences in self-regulation capacities, surely much could be learned from tracking decision times and latencies for such discrete affective auto-evaluation. These facts raise questions that can be answered only by future research. A careful assessment of the differences between aggressive expression and intensity of anger experience in terms of relative amount of decision time would be a more sensitive indicator of executive functions depletions and aggression-related increases than is an exclusive rating system.

Nevertheless, researchers have scarcely begun to study this area using a series of neuropsychological tests. We believe implications of research like the one presented here is essential and a necessary step for understanding the problem that are rooted in the study of aggression and its prevention planning. Our study allowed identification of negative feedback, even in the case of a task of no consequence, as a serious cause of poor executive performances and high aggressive tendencies. However, our data also showed that the individuals receiving a highly (success) or moderately (neutral) positive feedback performed significantly better and exhibited less aggressive tendencies. Thus, present research suggests the importance of contextual characteristics in dynamic relation between reactive and executive regulation of social behavior. By understanding those processes we should be in a better position to design an optimal training condition to improve executive capacities in children and adults from clinical as well as non-clinical populations. For example, application of such training program could have a synergic effect on patients and the hospital staff and it would be valuable for resolving major problems in health care section related to regulation of everyday social relations.
